# High-Precision Laser Time–Frequency Synchronization in Space Based on an Improved Kalman Filtering Method

**DOI:** 10.3390/s26113524

**Published:** 2026-06-02

**Authors:** Boao Sun, Xiaoqing Wang, Zhibin Sun, Fu Zheng

**Affiliations:** 1 National Space Science Center, Chinese Academy of Sciences, Beijing 100190, China; sundaren_1@163.com (B.S.); wangxq@nssc.ac.cn (X.W.); zbsun@nssc.ac.cn (Z.S.); 2 University of Chinese Academy of Sciences, Beijing 100049, China; 3 Center for Quantum Technology Research and Key Laboratory of Advanced Optoelectronic Quantum Architecture and Measurements (MOE), School of Physics, Beijing Institute of Technology, Beijing 100081, China

**Keywords:** time–frequency synchronization, free-space, space laser communication, laser ranging, Kalman filtering

## Abstract

To provide a ground-based experimental reference for free-space optical time–frequency synchronization in future space applications, this paper investigates the impact of beam drift and dynamic link-state variations on free-space laser links. A bidirectional free-space laser time–frequency synchronization and ranging system is established and the synchronization process is uniformly modeled. An improved Kalman filtering method based on innovation consistency is proposed in which a strong tracking mechanism enhances adaptability to model mismatch and abnormal observations; at the same time, an adaptive observation noise modeling strategy based on online statistical estimation characterizes the time-varying noise properties of free-space optical links. Experimental validation is conducted using an equivalent free-space laser link of approximately 321 m. The results show that the proposed method improves the time synchronization accuracy from 78.32 ps to 45.64 ps, corresponding to an enhancement of about 41%. In terms of time stability, the time deviation (TDEV) is reduced from $7.14\times 10^{-11}$ s to $4.33\times 10^{-11}$ s at an averaging time of τ=1 s, and from $4.20\times 10^{-12}$ s to $7.01\times 10^{-13}$ s at τ=800 s. For ranging performance, the system achieves an average measured distance of 321.56 m with a ranging standard deviation of 15.2 mm. These results demonstrate that the proposed approach enables high-precision and stable state estimation for integrated free-space laser time–frequency synchronization and ranging systems.

## 1. Introduction

High-precision time–frequency synchronization has become a key enabling technology for a wide range of space-based applications, including space gravitational wave detection, long-baseline interferometry, deep space formation flying, deep space tracking and control, navigation systems, and fundamental physics experiments [1,2,3]. Current time–frequency transfer techniques can be broadly classified into microwave-based and laser-based approaches. Owing to their high bandwidth, strong directionality, and excellent immunity to electromagnetic interference, laser links are capable of achieving picosecond- and even femtosecond-level synchronization accuracy [4,5,6,7]. Compared with conventional microwave links, laser-based techniques offer orders-of-magnitude improvements in stability and measurement precision, demonstrating significant potential in space–ground and inter-satellite links while providing essential technical support for navigation, constellation networking, and space science missions [8,9,10,11].

In recent years, extensive research efforts have been devoted to time–frequency synchronization over free-space laser links. Zhang et al. [12] realized an integrated scheme of bidirectional time synchronization and laser ranging by embedding timestamps into laser communication data and combining clock recovery with phase comparison techniques, achieving picosecond-level synchronization accuracy in equivalent space-link experiments. Xie et al. [13] proposed a bidirectional laser link-based approach in which time synchronization and distance information were jointly extracted through round-trip delay measurements, experimentally demonstrating nanosecond-level synchronization accuracy and meter-level ranging precision. Peng Zhang et al. [14] reduced link asymmetry through single-wavelength bidirectional transmission and high-power optical transceiver design, achieving picosecond-level time offset and long-term stability better than 20 ps over free-space distances of several tens of meters. Huang et al. [15] employed a femtosecond optical frequency comb at the master station and a continuous-wave laser at the slave station in combination with a bidirectional phase comparison mechanism to realize multi-site time–frequency synchronization over kilometer-scale fiber links and hundred-meter free-space links, effectively suppressing slow-varying link drifts to some extent. Despite these advances, existing studies have mainly focused on link architectures and hardware implementations [16,17,18], while systematic modeling and algorithmic optimization addressing beam drift, link-state variations, local oscillator frequency drift, and measurement uncertainties remain insufficient and require further investigation.

To mitigate the impact of frequency drift and measurement noise on synchronization performance, clock offset and frequency drift can be incorporated into a state-space model and dynamically estimated using Kalman filtering. However, conventional Kalman filters often suffer from insufficient tracking capability under fixed noise assumptions, when the measurement noise becomes time-varying, or when model mismatch and abrupt disturbances occur. Therefore, adaptive and robust Kalman filtering methods have been introduced into clock synchronization systems to improve estimation consistency and tracking robustness.

Zhang et al. [19] proposed a modified strong-tracking adaptive Kalman filter for IEEE1588 clock synchronization. Their method incorporates clock offset, network delay, frequency deviation, and network asymmetry into a multi-state model, while fuzzy logic, hypothesis testing, and a modified Sage–Husa covariance-estimation scheme are used to improve robustness against network delay jitter, asymmetry, clock drift, and measurement outliers. Li et al. [20] optimized synchronization message structures and transmission mechanisms by introducing fixed-length high-priority messages to reduce queuing and transmission uncertainties and further employed a multi-state Kalman filter to estimate clock offset and drift, reducing master–slave clock offset from the microsecond to nanosecond level in simulations. Peiji Li et al. [21] modeled clock offset and frequency drift as state variables and applied Kalman filtering to estimate timestamp uncertainty and link-delay jitter, achieving nanosecond-level synchronization while reducing hardware complexity and accelerating convergence.

In addition to these clock-synchronization-oriented studies, adaptive and robust Kalman filtering methods have also been widely investigated for systems with uncertain noise statistics, model mismatch, outliers, and non-Gaussian disturbances. Classical adaptive filtering methods such as the Sage–Husa adaptive filtering framework estimate unknown noise statistics online based on innovation or residual information, improving the applicability of Kalman filtering when the prior noise statistics are inaccurate or time-varying [22]. Covariance identification and covariance-matching techniques provide a theoretical basis for adjusting process and measurement noise covariance matrices according to the statistical properties of the innovation sequence [23]. More recently, variational Bayesian adaptive Kalman filters have been developed to recursively infer unknown or mismatched noise covariance matrices within a Bayesian framework [24]. Robust filtering frameworks based on variational Bayesian inference and generalized maximum-likelihood estimation have also been proposed to improve state estimation under uncertain measurement noise and abnormal observations [25]. In addition, adaptive robust variational Bayesian filtering methods have been further extended to heavy-tailed noise conditions, providing improved robustness against non-Gaussian disturbances and outliers [26].

Although the above studies demonstrate the effectiveness of adaptive covariance regulation and robust measurement updating, most existing filtering models for clock synchronization are developed for packet–network synchronization scenarios where the dominant uncertainties are network delay jitter, packet asymmetry, timestamp uncertainty, and clock drift. Therefore, their state variables and measurement models are usually constructed around clock offset, path delay, frequency drift, and link asymmetry. In contrast, the free-space laser time–frequency synchronization system considered in this work has a different measurement structure in which the measured synchronization output is mainly governed by the coupled evolution of time offset and frequency offset between two optical timing nodes, while the observations are affected by free-space optical link fluctuation, clock recovery jitter, received-power variation, optical terminal instability, and time-varying measurement uncertainty.

Unlike packet network-oriented synchronization models that explicitly describe path delay and link asymmetry, the free-space laser time–frequency synchronization system considered in this work is mainly characterized by the coupled evolution of time offset and frequency offset under optical link fluctuations. Therefore, the filtering model should be constructed according to this measurement structure and the fluctuation characteristics of the free-space laser link.

The novelty of this work lies in the application-oriented integration of adaptive and robust Kalman filtering concepts for free-space laser time–frequency synchronization. The proposed framework combines online estimation of observation noise with covariance regulation based on innovation consistency, allowing it to cope with time-varying optical link fluctuations, non-stationary measurement noise, and occasional abnormal observations in the measured 1PPS time-offset sequence. The main contribution is the adaptation, implementation, and experimental validation of this recursive filtering framework in a bidirectional free-space laser time–frequency synchronization system.

## 2. Free-Space Laser Synchronization and Ranging System

### 2.1. Free-Space Laser Time–Frequency Synchronization System

**Figure 1:**
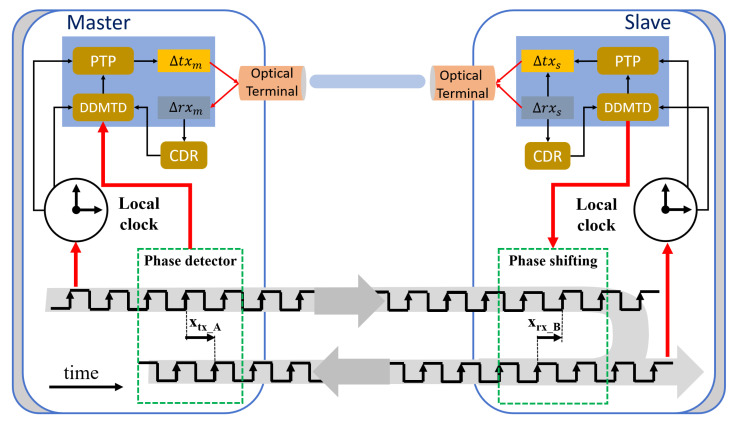
System architecture of the free-space laser time–frequency synchronization experiment.

As shown in Figure 1, a bidirectional free-space laser time–frequency synchronization system is established in this work. The system consists of a master node and a slave node, which exchange time and frequency information through a bidirectional free-space optical link. The master and slave nodes share identical hardware architectures and functional modules, including a local clock, a time measurement unit, and an optical terminal, while operating under an asymmetric master–slave clock control scheme.

At each node, the local clock serves as the local estimate of the global time reference and provides a unified timing reference for time tagging, phase measurement, and physical-layer clock derivation. The time measurement unit comprises a precision time protocol (PTP) module and a digital dual-mixer time difference (DDMTD) phase detector. The PTP module is responsible for estimating link delay and time offset based on exchanged timing information, whereas the DDMTD module performs high-resolution phase difference measurements between the recovered clock and the local clock.

The optical terminals at the master and slave nodes enable bidirectional laser communication by collimating, transmitting, and receiving optical beams, allowing time and frequency information to propagate back and forth between the two nodes. At the master node, the local clock drives the physical-layer transmit clock, which is modulated onto the optical carrier and transmitted to the slave node via the free-space optical link. At the slave node, the received optical signal is converted into an electrical signal and fed into a clock data recovery (CDR) module to extract the physical-layer received clock from the communication signal.

Subsequently, the DDMTD module measures the phase difference between the recovered clock and the local clock at the slave node. Through a continuous phase-shifting mechanism, the local clock is gradually adjusted to achieve phase alignment with the received clock. By means of this bidirectional exchange and phase alignment process, end-to-end frequency transfer between the master and slave nodes is realized, after which the corresponding time offset information is extracted to support high-precision time synchronization.

**Figure 2:**
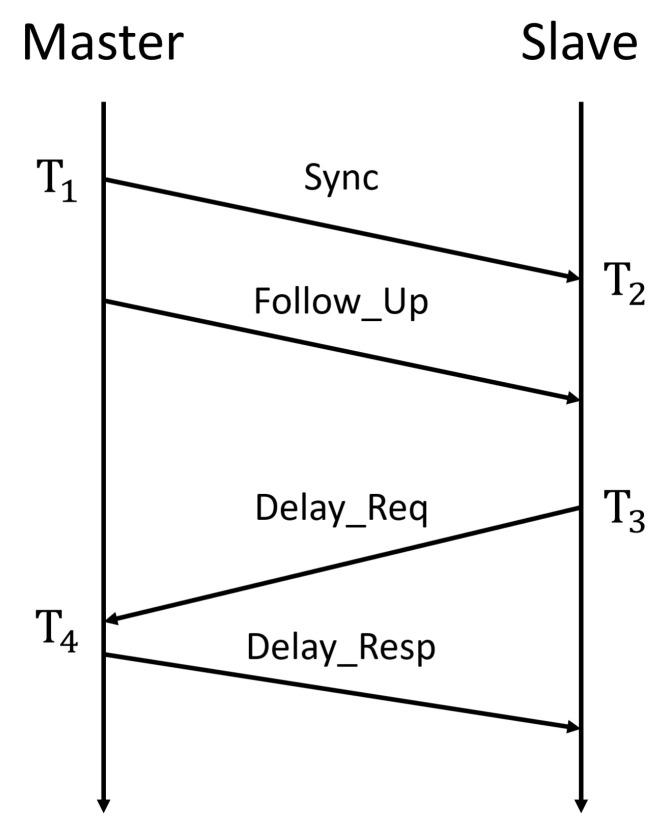
Master–slave synchronization process.

The PTP synchronization procedure is illustrated in Figure 2. The master clock transmits a Sync message to the slave clock and records the transmission timestamp $t_{1}$, which is subsequently conveyed to the slave clock in the corresponding Follow_Up message. Upon reception of the Sync message, the slave clock records the arrival timestamp $t_{2}$. The slave clock then sends a Delay_Request message and records the transmission timestamp $t_{3}$. After receiving this message, the master clock records the reception timestamp $t_{4}$ and returns it to the slave clock via a Delay_Response message.

Let the time offset between the master clock and the slave clock at a given synchronization instant be denoted as $\theta_{1}$, and let the one-way propagation delay from the master to the slave be $\tau_{MS}$. The relationship between the timestamps $t_{1}$ and $t_{2}$ can then be expressed as

(1) $$t_{2}=t_{1}+\theta_{1}+\tau_{MS}.$$

Similarly, at the subsequent synchronization instant, let the time offset between the master and slave clocks be $\theta_{2}$, and let the propagation delay from the slave to the master be $\tau_{SM}$. The timestamps $t_{3}$ and $t_{4}$ satisfy

(2) $$t_{4}=t_{3}+\theta_{2}+\tau_{SM}.$$

It is further assumed that the clock offset remains unchanged within a single synchronization cycle

(3) $$\theta_{1}=\theta_{2}=\theta ,$$

and that the forward and backward propagation delays are equal:

(4) $$\tau_{MS}=\tau_{SM}=\tau .$$

Under these assumptions, the one-way propagation delay can be estimated as

(5) $$\tau =\frac{\left(t_{2}-t_{1}\right)+\left(t_{4}-t_{3}\right)}{2},$$

while the clock offset between the master and slave clocks can be obtained as

(6) $$\theta =t_{1}-t_{2}+\tau .$$

Accordingly, the slave clock can in principle synchronize its local time to the master clock using the acquired timestamps $t_{1}$, $t_{2}$, $t_{3}$, and $t_{4}$.

To achieve high-resolution phase alignment, a digital dual-mixer time difference (DDMTD) module is employed to detect the phase difference between the master and slave clocks. By scaling the picosecond-level phase difference between the local clock and the recovered data clock to the nanosecond level or beyond, the DDMTD enables accurate sampling using a low-frequency reference clock. The detected phase error is then used by a phase adjustment module at the slave node to finely tune the local clock, achieving precise synchronization with the master clock.

By appropriately combining the timestamp information, the influence of clock offset on propagation delay estimation can be eliminated. The round-trip propagation delay is defined as

(7) $$\tau_{\mathrm{RT}}=\left(t_{2}-t_{1}\right)+\left(t_{4}-t_{3}\right).$$

Under ideal symmetric-link conditions, the round-trip delay and one-way propagation delay satisfy

(8) $$\tau =\frac{\tau_{\mathrm{RT}}}{2}.$$

Consequently, the spatial distance *d* between the master and slave nodes can be expressed as

(9) $$d=\frac{c\;\tau_{\mathrm{RT}}}{2},$$

where *c* denotes the speed of light in free space.

In practical free-space environments, however, the estimated round-trip delay inevitably exhibits random fluctuations due to link-state variations and time measurement noise.

### 2.2. State-Space Model

In free-space laser time–frequency synchronization, the temporal relationship between two terminal nodes is primarily determined by the clock time offset and frequency offset, both of which exhibit pronounced dynamic characteristics. Meanwhile, the measurement results are inevitably affected by clock noise, link disturbances, and uncertainties in the measurement system. To enable dynamic estimation of the synchronization process and provide a unified mathematical framework for subsequent design of the improved Kalman filtering algorithm, the problem is modeled as a discrete-time state-space system.

Considering the clock relationship between two nodes connected by a bidirectional free-space laser link, the system state vector at the *k*-th discrete time instant is defined as

(10) $$x_{k}=\left[\begin{array}{c} \theta_{k}\\ \alpha_{k} \end{array}\right],$$

where $\theta_{k}$ denotes the time offset between the two nodes and $\alpha_{k}$ represents the frequency offset between their local clocks. These two state variables jointly characterize the dominant dynamics of the free-space laser time–frequency synchronization process. Specifically, the time offset accumulates as the integral of the frequency offset over time, while the frequency offset typically evolves slowly.

Under discrete-time conditions, assuming that the frequency offset varies slowly within adjacent sampling intervals and that the evolution of the time offset is dominated by the frequency offset, the state transition equation can be expressed as

(11) $$x_{k}=Fx_{k-1}+\omega_{k},$$

where F denotes the state transition matrix, given by

(12) $$F=\left[\begin{array}{cc} 1 & T\\ 0 & p \end{array}\right].$$

Here, *T* is the sampling interval and p∈(0,1] is a decay factor associated with the frequency-offset state, which is introduced to describe the slow-varying or weakly damped evolution of the clock frequency offset. We tested several nearby values of *p* and observed that when *p* was set too small, the estimated frequency-offset state became over-damped, which weakened the model’s ability to follow slow clock-drift variations. In contrast, when *p* was set too close to 1, the recursive estimation became more prone to long-term accumulation of frequency-offset fluctuations. Under the present experimental conditions, p=0.998 provided a better compromise, yielding a smoother state evolution while maintaining lower instability over long averaging times.

In Equation (11), $\omega_{k}$ denotes the process noise vector, which accounts for the unmodeled slow variations of the time-offset and frequency-offset states. It is assumed to be a zero-mean random process with covariance matrix Q, i.e.,

(13) $$\omega_{k}∼N\left(0,Q\right).$$

Since the state vector consists of the time offset and the frequency offset, the process noise covariance matrix is set as

(14) $$Q=\left[\begin{array}{cc} Q_{\theta } & 0\\ 0 & Q_{\alpha } \end{array}\right],$$

where $Q_{\theta }$ and $Q_{\alpha }$ denote the process-noise variances associated with the time-offset and frequency-offset states, respectively. The off-diagonal terms of Q are set to zero because the deterministic coupling between the time offset and the frequency offset has already been described by the state-transition matrix F.

The values of $Q_{\theta }$ and $Q_{\alpha }$ determine the confidence assigned to the state-transition model. If the process noise covariance is too small, the filter relies excessively on the model prediction and may not follow slow clock-drift variations or link-state changes effectively; conversely, if it is too large, the filter becomes overly sensitive to measurement fluctuations and may introduce additional residual jitter. In this work, $Q_{\theta }=1.0\times 10^{-28}$ and $Q_{\alpha }=5.0\times 10^{-27}$ are adopted according to preliminary tests on the recorded experimental data and the expected slow-varying behavior of the synchronization state.

In the bidirectional free-space laser link, observations related to the clock offset and propagation delay can be obtained through timestamp exchange or round-trip delay measurements. The observation vector at time instant *k* is defined as

(15) $$z_{k}=\left[\begin{array}{c} \theta_{k}^{\left(m\right)}\\ \alpha_{k}^{\left(m\right)} \end{array}\right]$$

and the corresponding observation equation can be written as

(16) $$z_{k}=Hx_{k}+v_{k},$$

where $H=I_{2}$ is the observation matrix and $v_{k}$ denotes the observation noise vector.

Specifically, the frequency-offset observation is approximated from adjacent time-offset measurements as

(17) $$\alpha_{k}^{\left(m\right)}\approx \frac{\theta_{k}^{\left(m\right)}-\theta_{k-1}^{\left(m\right)}}{T};$$

as a result, its measurement error is statistically correlated with the measurement error of the time offset $\theta_{k}^{\left(m\right)}$. Consequently, the observation noise covariance matrix is no longer diagonal. For a given time-offset measurement noise variance $\sigma_{\theta }^{2}$, the covariance structure can be expressed as

(18) $$R=\left[\begin{array}{cc} \sigma_{\theta }^{2} & \frac{\sigma_{\theta }^{2}}{T}\\ \frac{\sigma_{\theta }^{2}}{T} & \frac{\sigma_{\theta }^{2}}{T^{2}} \end{array}\right],$$

where $\sigma_{\theta }^{2}$ denotes the variance of the measurement noise of the time offset. This modeling approach more accurately reflects the coupling between time and frequency measurement errors in practical systems.

From the above state-space formulation, the free-space laser time–frequency synchronization problem can be regarded as a joint state estimation problem involving time offset and frequency offset. The model is compact, physically interpretable, and well suited for online estimation and real-time processing.

However, in practical free-space laser links, the measurement noise often exhibits time-varying and non-Gaussian characteristics, and system model parameters may vary with environmental conditions and operating states. Under fixed assumptions for process noise and observation noise, conventional Kalman filtering methods suffer from limitations in estimation accuracy and long-term stability. Therefore, it is necessary to introduce improved Kalman filtering strategies that explicitly address noise variation and model mismatch on the basis of the above model in order to enhance robustness and estimation performance under complex and time-varying link conditions.

## 3. Improved Kalman Filtering Algorithm

To address the limitations of conventional Kalman filtering in handling time-varying and non-Gaussian measurement disturbances, this paper proposes an improved Kalman filtering method tailored for free-space laser time–frequency synchronization. While preserving the basic Kalman filtering framework, the proposed approach incorporates a strong tracking mechanism based on innovation consistency and an adaptive observation noise strategy based on online statistical estimation, which together enhancing robustness and stability under complex and time-varying link conditions.

In conventional Kalman filtering, the filter gain is jointly determined by the predicted error covariance and observation noise covariance. When the assumed state-space model does not fully match the actual system evolution or when abrupt disturbances appear in the measurements, a fixed covariance recursion may lead to insufficient responsiveness to newly arrived observations. In order to improve robustness under such conditions, in this work, an innovation-consistency-based strong-tracking mechanism is introduced.

For the predicted state ${\hat{x}}_{k}^{-}$, the innovation vector is defined as

(19) $$\nu_{k}=z_{k}-H{\hat{x}}_{k}^{-}.$$

Substituting the observation equation in (16) into Equation (19) yields

(20) $$\nu_{k}=H\left(x_{k}-{\hat{x}}_{k}^{-}\right)+v_{k}.$$

Letting $e_{k}^{-}=x_{k}-{\hat{x}}_{k}^{-}$ denote the predicted state error, the innovation covariance can be written as

(21) $$S_{k}=E\left[\nu_{k}\nu_{k}^{T}\right]=HE\left[e_{k}^{-}e_{k}^{-T}\right]H^{T}+HE\left[e_{k}^{-}v_{k}^{T}\right]+E\left[v_{k}e_{k}^{-T}\right]H^{T}+E\left[v_{k}v_{k}^{T}\right].$$

Under the standard assumption that the predicted state error and the observation noise are mutually independent, the cross-terms vanish, i.e.,

(22) $$E[e_{k}^{-}v_{k}^{T}]=0.$$

Therefore, the innovation covariance is obtained as

(23) $$S_{k}=HP_{k}^{-}H^{T}+R_{k},$$

where $P_{k}^{-}=E\left[e_{k}^{-}e_{k}^{-T}\right]$ and $R_{k}=E\left[v_{k}v_{k}^{T}\right]$. In this work, $R_{k}$ is not assumed to be diagonal, as the frequency-offset observation is derived from adjacent time-offset measurements and as such is statistically correlated with the time-offset observation error, as discussed in Section 2.2.

Based on the innovation vector and its covariance, the normalized innovation squared (NIS) is defined as

(24) $${\mathrm{NIS}}_{k}=\nu_{k}^{T}S_{k}^{-1}\nu_{k}.$$

The NIS serves as an indicator of innovation consistency that quantitatively measures whether the current innovation remains within the statistically expected range determined by the assumed model and noise covariance. In physical terms, it reflects whether the discrepancy between the current observation and the predicted observation can still be explained by the nominal state-space model and the assumed measurement uncertainty. A relatively small NIS indicates that the innovation is statistically consistent with the model, whereas an excessively large NIS suggests model mismatch, transient link disturbance, or abnormal observation deviation.

For a Gaussian innovation sequence under a correctly specified model, ${\mathrm{NIS}}_{k}$ approximately follows a chi-square distribution with degrees of freedom equal to the dimension of the observation vector. Since the observation vector in this work is two-dimensional, a chi-square test with two degrees of freedom is adopted. In the implementation, a 95% confidence level is used, corresponding to the threshold

(25) $$\chi_{0.95,2}^{2}=5.991.$$

When ${\mathrm{NIS}}_{k}\leq \chi_{0.95,2}^{2}$, the innovation is regarded as statistically consistent and the nominal covariance prediction is preserved; when ${\mathrm{NIS}}_{k}>\chi_{0.95,2}^{2}$, the innovation is considered inconsistent and the strong-tracking mechanism is activated.

Specifically, the predicted error covariance is adaptively inflated as

(26) $$P_{k}^{-}\leftarrow \lambda_{k}P_{k}^{-},$$

where $\lambda_{k}\geq 1$ is not fixed but determined according to the exceedance degree of the NIS:

(27) $$\lambda_{k}=1+\gamma \max\left(0,\frac{{\mathrm{NIS}}_{k}}{\chi_{0.95,2}^{2}}-1\right).$$

In this work, γ=0.10 is used as the fading-strength parameter and the upper bound of the covariance inflation factor is set to $\lambda_{\max}=10$; accordingly, $\lambda_{k}$ increases continuously with the exceedance degree of the NIS, rather than being assigned a fixed inflation value. This design allows for mild covariance inflation when the innovation inconsistency is limited while providing stronger responsiveness under pronounced innovation growth. The upper bound is introduced to avoid excessive covariance expansion and possible numerical instability. Therefore, the NIS is not only used as a statistical diagnostic quantity but also serves as the direct decision variable for triggering and regulating the strength of the strong tracking mechanism.

In practical free-space laser time–frequency synchronization experiments, the observation noise level varies with link state and environmental conditions; therefore, instead of using a fixed observation-noise variance, this work employs an exponentially weighted recursive estimator to update the time-offset measurement statistics online. Let ${\bar{\theta }}_{k}$ denote the exponentially weighted recursive mean of the time-offset observations. It is updated as

(28) $${\bar{\theta }}_{k}={\bar{\theta }}_{k-1}+\beta \left(\theta_{k}^{\left(m\right)}-{\bar{\theta }}_{k-1}\right),$$

where β∈(0,1) is the smoothing factor. Based on this recursive mean, the corresponding variance estimate is updated by

(29) $${\hat{\sigma }}_{\theta ,k}^{2}=\left(1-\beta \right)\left[{\hat{\sigma }}_{\theta ,k-1}^{2}+\beta {\left(\theta_{k}^{\left(m\right)}-{\bar{\theta }}_{k-1}\right)}^{2}\right],$$

where ${\hat{\sigma }}_{\theta ,k}^{2}$ denotes the online estimate of the time-offset measurement noise variance at the *k*-th sampling instant. This form is equivalent to an exponentially weighted recursive variance estimator rather than a sliding window-based average.

In the experiments, β=0.30 is used as a compromise between sensitivity to short-term noise variation and robustness against isolated fluctuations. Larger β would make the recursive variance estimate more sensitive to instantaneous disturbances, whereas a smaller β would slow down its adaptation to time-varying link conditions.

The estimated variance is then substituted into the covariance structure introduced in Section 2.2 to construct the time-varying correlated observation noise covariance matrix $R_{k}$, enabling adaptive adjustment of measurement weighting in real time:

(30) $$R_{k}=\left[\begin{array}{cc} {\hat{\sigma }}_{\theta ,k}^{2} & \frac{{\hat{\sigma }}_{\theta ,k}^{2}}{T}\\ \frac{{\hat{\sigma }}_{\theta ,k}^{2}}{T} & \frac{{\hat{\sigma }}_{\theta ,k}^{2}}{T^{2}} \end{array}\right]$$

where ${\hat{\sigma }}_{\theta ,k}^{2}$ denotes the online estimate of the time-offset measurement noise variance at the *k*-th sampling instant.

The main implementation parameters of the proposed filter are summarized in Table 1. These parameters were fixed during the real-time experimental operation. The process noise covariance parameters $Q_{\theta }$ and $Q_{\alpha }$ were selected according to the expected slow-varying behavior of the time-offset and frequency-offset states. The smoothing factor β controls the update rate of the online observation noise estimator, while the fading-strength parameter γ controls the intensity of the innovation consistency-based covariance regulation. In this work, the parameters are selected to ensure stable recursive operation and balanced response to time-varying link fluctuations rather than to optimize a single offline data segment.

Based on the above state-space model, innovation consistency evaluation, and adaptive observation noise estimation strategy, the computational procedure of the proposed method is summarized in Table 2.

**Figure 3:**
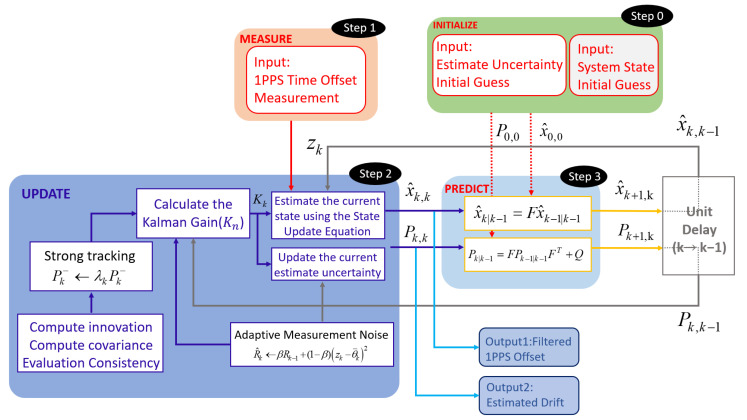
Overall algorithm structure and information flow.

The overall structure and information flow of the proposed algorithm are illustrated in Figure 3. In the figure, the uncertainty of state estimation is represented by the covariance propagation and update process; in particular, the predicted error covariance describes the uncertainty of one-step state prediction, while the innovation covariance characterizes the expected statistical dispersion of innovation in the observation space. These quantities provide the basis for evaluating the innovation consistency and for subsequent covariance adjustment in the strong tracking mechanism. The core idea is to retain the prediction–update framework of Kalman filtering while introducing innovation consistency-based online monitoring and adaptive regulation mechanisms, thereby forming a closed-loop adaptive filtering process.

Within each sampling interval, the algorithm first assigns initial values to the system state and estimation error covariance during the initialization stage, providing prior conditions for subsequent recursion. In the measurement stage, the system acquires the 1PPS time offset observation at the current time instant and uses it as the input for the filtering update. Based on the predicted state from the previous time step, the innovation is computed as the difference between the current observation and its prediction, representing the new information introduced by the measurement.

The algorithm then enters the update stage, where the statistical properties of the innovation are analyzed. The normalized innovation squared (NIS) is calculated and employed as a consistency evaluation metric. When the innovation statistics deviate from their theoretical expectations, this indicates a mismatch between the assumed system model or noise statistics and the actual state evolution. In this case, the strong tracking mechanism is activated to adaptively inflate the predicted error covariance, enhancing the filter responsiveness to new measurement information; conversely, when innovation consistency is maintained, the original predicted covariance is preserved to avoid unnecessary estimation perturbations.

Meanwhile, considering the pronounced time-varying and non-Gaussian characteristics of observation noise in free-space laser links, the algorithm estimates the observation noise level through online statistical analysis and dynamically updates the observation noise covariance matrix. This adaptive noise modeling process operates in conjunction with the evaluation of innovation consistency, enabling the filter to adjust the weighting of measurement information according to real-time observation quality in order to improve the overall estimation stability.

After covariance adjustment and observation noise update, the algorithm performs a standard Kalman filtering update to obtain the current estimates of the time offset and frequency offset. Finally, the updated state estimate and error covariance are propagated to the next sampling interval for state prediction, forming a closed-loop recursive estimation process.

## 4. Experimental Results and Discussion

In this section, the experimental results of the free-space laser time–frequency synchronization system are presented. First, the real-time filtering performance is analyzed. The proposed filter was implemented in the synchronization data processing program and operated recursively during data acquisition; therefore, the filtered time-offset sequence reported in Section 4.3 corresponds to the real-time filtering output. Then, an offline recursive comparison with representative Kalman filtering-based methods is conducted to further evaluate the relative ability of the proposed method to suppress fluctuations. Finally, the ranging performance of the system is analyzed.

### 4.1. Free-Space Experimental System Setup

**Figure 4:**
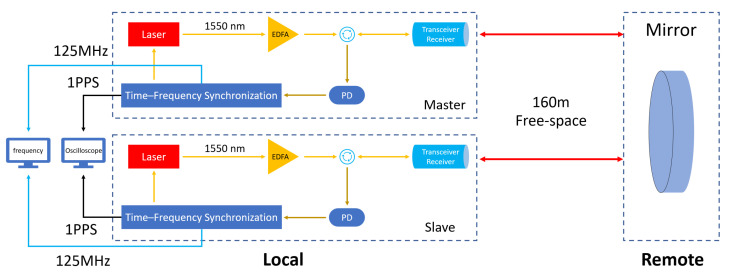
Schematic diagram of the experimental system.

To verify the effectiveness of the proposed improved Kalman filtering algorithm under practical free-space laser link conditions, a master–slave free-space laser time–frequency synchronization experimental system was established, as illustrated in Figure 4. The system consists of two optical communication terminals, namely, the master node and slave node, which exchange time and frequency information through a bidirectional free-space laser link.

At each terminal, an integrated laser source is employed as the optical carrier, generating a continuous-wave signal with a central wavelength of 1550 nm. The optical signal is amplified by an erbium-doped fiber amplifier (EDFA) and transmitted into the free-space link via the optical transceiver front-end. The system operates on a 1 Gbps high-speed optical communication link for data transmission, providing sufficient bandwidth for the exchange of timing and synchronization information. The path of free-space propagation has a length of approximately 160 m. A reflector is placed at the remote end to permit the return of the optical signal, forming a bidirectional free-space propagation link. The returned optical signal is coupled into the optical receiving module and converted into an electrical signal by a photodetector (PD).

After photoelectric conversion, the received signal is fed into the time–frequency synchronization module, which integrates clock and data recovery (CDR) and time synchronization processing functions. This module recovers the synchronized clock from the received signal and extracts time and phase offset information. Time and frequency alignment between the local clock and received clock is achieved through continuous phase adjustment of the recovered clock to complete the master–slave time–frequency synchronization process.

The master and slave nodes are each equipped with an independent board-level 125 MHz crystal oscillator as the local clock reference. The two oscillators are not derived from a common external reference during the free-space time-transfer experiment. Therefore, the experimental system operates with two independently clocked timing nodes rather than with two nodes sharing the same reference clock. The independent stability of the local clock references was evaluated before the synchronization experiment. The measured Allan deviation of the master-side local clock was approximately $1.02\times 10^{-8}$ at τ=1 s and $8.75\times 10^{-10}$ at τ=1000 s, while that of the slave-side local clock was approximately $1.77\times 10^{-10}$ at τ=1 s and $8.33\times 10^{-10}$ at τ=1000 s. These measurements were used to verify the basic stability of the local clock references before the synchronization experiment.

At both the master and slave nodes, the time–frequency synchronization module outputs a one-pulse-per-second (1PPS) signal and a 125 MHz frequency synchronization signal. The 1PPS signals from the two nodes are connected to a TDS 7404 oscilloscope to measure the time offset between the master and slave clocks, while the 125 MHz signals are fed into an Agilent 53220 frequency counter to evaluate the frequency stability of the synchronized clocks.

**Figure 5:**
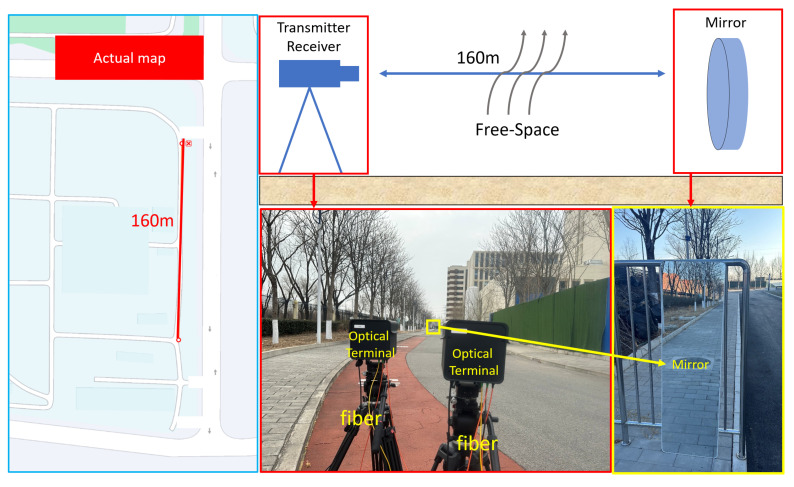
Experimental scenario and free-space link configuration.

The experimental scenario and free-space link configuration are illustrated in Figure 5. The experiment was conducted in an outdoor open environment in which the optical terminals at the master and slave nodes were deployed along a roadway with a straight-line separation of approximately 160 m. To establish a bidirectional free-space transmission link, a reflector was placed at the remote end to reflect the transmitted optical signal back toward the transmitting terminal, thereby forming a round-trip propagation path.

The left panel of Figure 5 presents the physical length of the free-space link measured using map-based distance calibration, which serves as a reference for the actual propagation distance. The right panel shows on-site photographs of the optical terminals and the reflector, illustrating their practical installation conditions. Both the master and slave optical communication terminals were connected to local electronic processing modules via optical fibers. The optical terminals were mounted on tripods and manually aligned to establish and maintain the free-space laser link.

### 4.2. Timing Error Budget of the Calibrated Measurement Chain

The externally measured 1PPS time offset is used as the main performance indicator in this work. This quantity reflects the combined influence of the calibrated time transfer chain, free-space optical link, electronic circuits, clock recovery process, residual clock-induced fluctuation, and external measurement instrument. Before the experiment, deterministic internal delays and fixed link-delay terms were compensated through system calibration. Therefore, these deterministic terms mainly affect the absolute offset bias before calibration and are not the direct target of the proposed filtering method. After calibration, the remaining standard deviation of the measured 1PPS offset is mainly determined by random and time-varying components such as 1PPS output jitter, residual clock-induced fluctuation, clock recovery jitter, electronic circuit noise, free-space optical link fluctuation, and external measurement noise.

To clarify the physical origin of the measured timing fluctuation and the role of the proposed filtering method, the approximate timing error budget related to the measured 1PPS offset is summarized in Table 3.

As shown in Table 3, the reported standard deviation of the 1PPS time offset is mainly affected by residual random and time-varying components after system calibration. The proposed filtering method does not change the calibrated fixed delay terms or physically reduce the intrinsic noise of the local clock source or the hardware output jitter. Instead, it estimates the time-offset state from noisy observations and suppresses the influence of residual time-varying disturbances in the measured 1PPS offset sequence. Therefore, the filtered result should be interpreted as an improvement in the stability of the externally measured 1PPS synchronization output under the calibrated experimental configuration rather than as a reduction of deterministic hardware delays.

### 4.3. Real-Time Experimental Filtering Results

**Figure 6:**
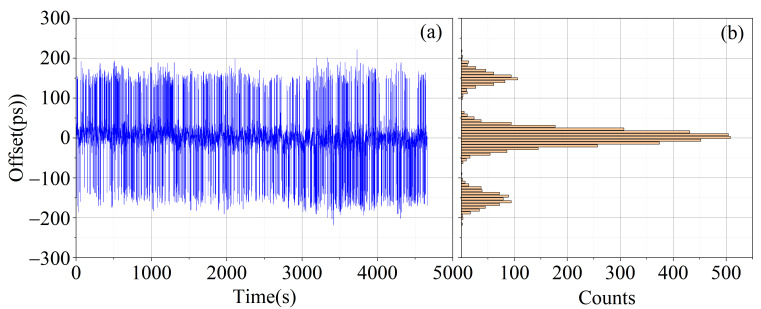
Raw 1PPS time offset measurement results: (**a**) time-domain sequence of the 1PPS time offset and (**b**) histogram of the measured 1PPS time offset.

The filtering results presented in this subsection were obtained from the real-time implementation of the proposed algorithm during the experiment. At each sampling instant, the measured 1PPS time offset was processed recursively by the filter, then the filtered output was recorded together with the raw measurement sequence. The experimental results record the time offset sequence of the one-pulse-per-second (1PPS) signals between the master and slave nodes, which characterizes the instantaneous time synchronization performance under free-space laser link conditions. As shown in Figure 6a, the raw 1PPS time offset sequence is plotted as a function of the sampling time. From the temporal characteristics, the 1PPS offset exhibits pronounced random fluctuations on short time scales; its statistical distribution suggests the presence of multiple measurement states and intermittent disturbances over the observation interval.

To further analyze the statistical properties of the 1PPS time offset, the corresponding histogram is presented in Figure 6b. It can be observed that the offset distribution deviates significantly from a unimodal Gaussian shape. In addition to the main distribution region, several secondary distribution components are present, indicating the superposition of multiple noise states or intermittent disturbances in the measurement data. Statistical analysis shows that the standard deviation of the raw 1PPS time offset sequence is approximately 78.32 ps.

In the free-space laser time–frequency synchronization experiment, the phase difference between the local clocks at the master and slave nodes reflects not only the cumulative effect of frequency offset but also random fluctuations introduced by laser link disturbances, local oscillator phase noise, and environmental factors. To further investigate the statistical characteristics of this phase difference, both time-domain and probabilistic analyses were performed on the measured 125 MHz clock phase data obtained from the experiment.

**Figure 7:**
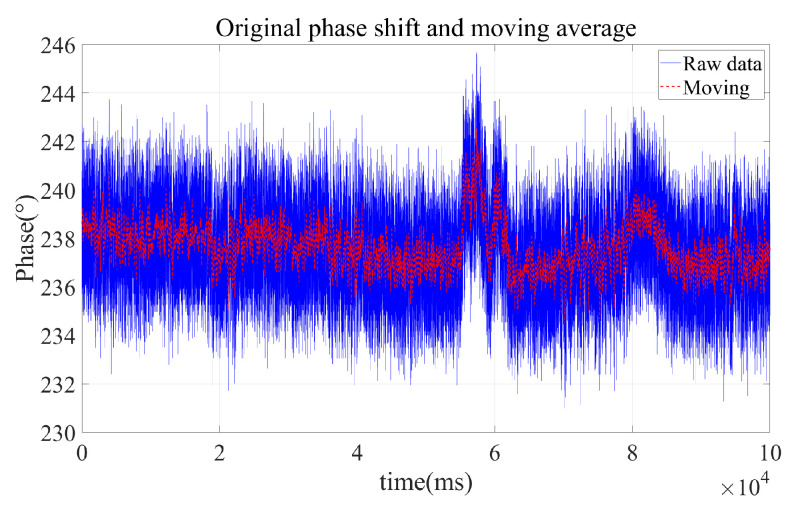
Time-domain evolution of the measured 125 MHz clock phase offset. The solid curve represents the raw phase offset, while the dashed curve shows the corresponding moving-average trend.

**Figure 8:**
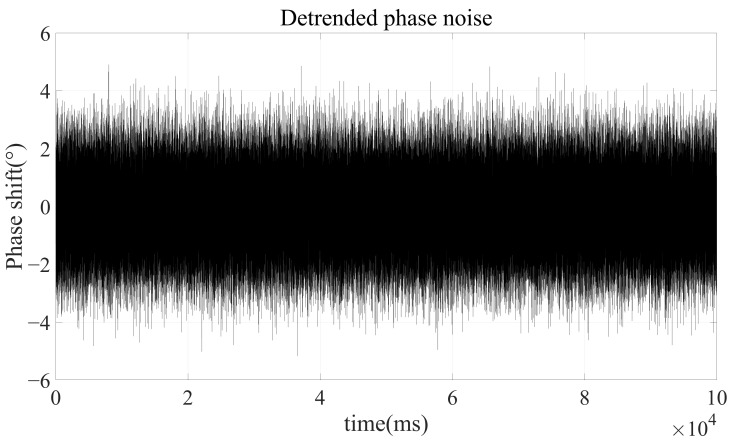
Detrended phase offset sequence of the 125 MHz clock after removing the slowly varying trend component.

Figure 7 shows the temporal evolution of the raw phase offset together with its moving-average curve. It can be observed that the phase difference exhibits a slowly varying trend component over time, superimposed with pronounced high-frequency random fluctuations. After removing the trend from the raw phase data, the resulting phase offset sequence is shown in Figure 8. The detrended phase sequence has a mean value close to zero and does not exhibit significant periodic components, indicating that, over the considered time scale, the residual phase noise can be reasonably approximated as a stationary random process. It should be noted that the detrending operation shown in Figure 8 was performed in postprocessing for statistical analysis and visualization of the measured phase data, and was not part of the real-time synchronization or filtering process.

**Figure 9:**
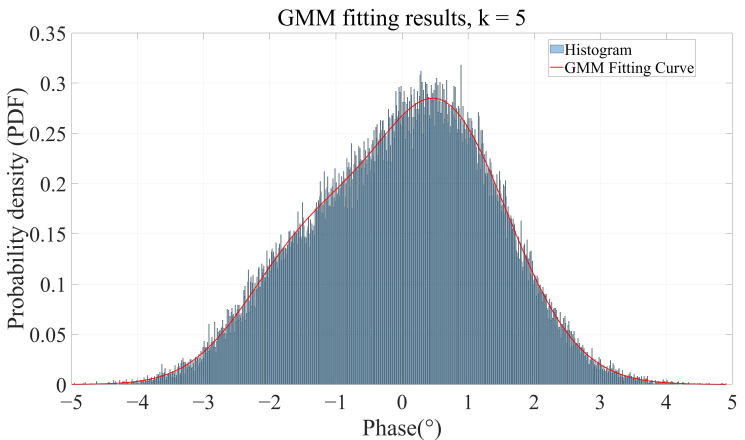
Probability distribution of the detrended phase noise and Gaussian mixture model (GMM) fitting results.

Further statistical analysis was performed on the detrended phase noise by evaluating its probability distribution and applying Gaussian mixture model (GMM) fitting, as shown in Figure 9. The fitting results indicate that the phase noise can be well represented by a superposition of five Gaussian components, with component standard deviations concentrated in the range of approximately 0.85–1.17°. The corresponding statistical parameters are summarized in Table 4.

These results demonstrate that the phase noise exhibits pronounced multimodal and heavy-tailed characteristics, making it difficult for a single Gaussian model to adequately capture its distribution. Meanwhile, the weighted mean of the fitted GMM is close to zero (approximately −0.0008°), which is consistent with the zero-mean assumption after detrending. The overall equivalent standard deviation is approximately 1.38°, reflecting the aggregate fluctuation level of the phase noise under free-space laser link conditions.

**Figure 10:**
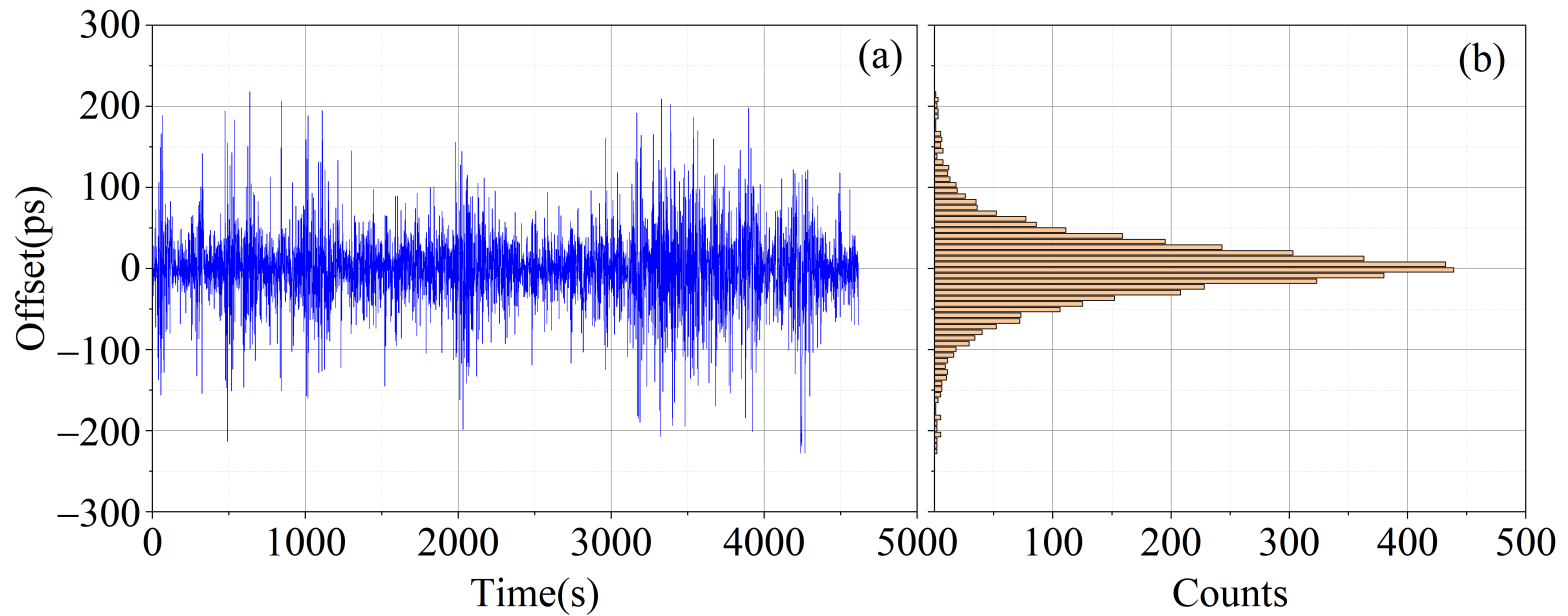
Filtered 1PPS time offset results after applying the proposed improved Kalman filtering algorithm: (**a**) time-domain sequence of the filtered 1PPS time offset and (**b**) histogram of the filtered 1PPS time offset.

The real-time filtered 1PPS time-offset results obtained by the proposed improved Kalman filtering algorithm are shown in Figure 10, including both the filtered time-offset sequence and its statistical distribution. From the time-domain results, it can be observed that the overall fluctuation amplitude of the 1PPS time offset is significantly reduced after filtering. Compared with the frequent abrupt offset excursions observed in the raw data, the filtered time offset sequence exhibits a smoother and more continuous evolution, with only limited residual disturbances occurring at a few time instants.

From a statistical perspective, the histogram of the filtered 1PPS time offset shows a clear unimodal distribution, with the majority of samples concentrated around the mean value. The distribution is noticeably more compact than that of the raw data. Quantitatively, the standard deviation of the 1PPS time offset is reduced from approximately 78.32 ps in the original measurements to about 45.64 ps after filtering, indicating a substantial reduction in synchronization jitter and a pronounced contraction of the overall distribution range. These results demonstrate that, under complex free-space laser link conditions, the proposed improved Kalman filtering algorithm effectively enhances the stability of 1PPS time synchronization while preserving the system dynamic responsiveness.

**Figure 11:**
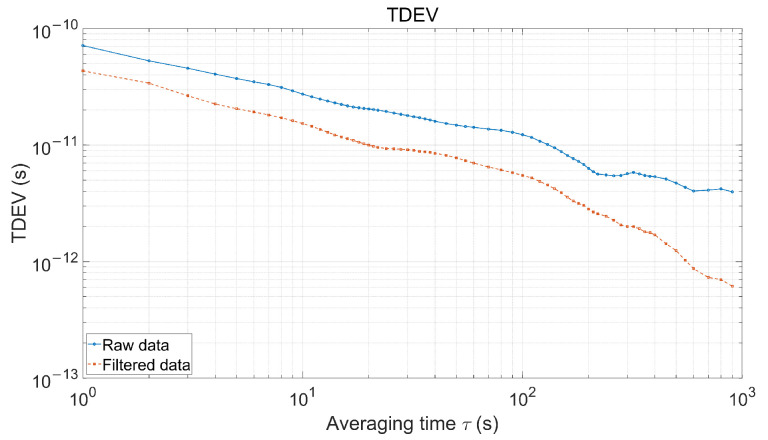
Comparison of time deviation (TDEV) of the 1PPS time offset before and after applying the proposed improved Kalman filtering algorithm.

To evaluate the impact of the proposed improved Kalman filtering algorithm on time synchronization stability over different time scales, the time deviation (TDEV) of the 1PPS time offset sequences was calculated for both the raw and filtered data. Figure 11 presents a comparison of TDEV as a function of the averaging time τ under the two conditions. The sampling interval was set to 1 s, and the total observation duration was approximately 1 h.

After applying the proposed improved Kalman filtering algorithm, the TDEV curve exhibits a clear reduction over both short and long time scales. Specifically, the TDEV of the time offset before filtering is approximately $7.14\times 10^{-11}$ s at an averaging time of τ=1 s, which is reduced to $4.33\times 10^{-11}$ s after filtering. At a longer averaging time of τ=800 s, the TDEV decreases from $4.20\times 10^{-12}$ s to $7.01\times 10^{-13}$ s, indicating a substantial improvement in time stability. These results indicate that the proposed filtering method not only suppresses short-term synchronization jitter but also improves time synchronization stability over the tested averaging-time range.

### 4.4. Offline Comparison with Representative Kalman Filtering Methods

To further evaluate the proposed filtering method against representative Kalman filtering-based approaches, an offline recursive comparison was conducted using the same measured 1PPS time-offset sequence. The compared methods include the standard Kalman filter, variational Bayesian Kalman filter (VB-KF), M-estimation-based robust Kalman filter, and the proposed method.

For a fair comparison, all of the Kalman filtering-based methods were implemented in the same causal recursive framework and were used to process the same measured 1PPS time-offset sequence. The same state model, sampling interval, process noise covariance, and initial covariance were adopted for all compared methods. The state vector was defined as $x_{k}={\left[\theta_{k},\alpha_{k}\right]}^{T}$, where $\theta_{k}$ denotes the time offset and $\alpha_{k}$ denotes the state related to the frequency offset. The sampling interval was set to T=1 s, while the decay factor for the frequency offset was set to p=0.998. The process noise covariance parameters were kept consistent with those used in the proposed method.

For the VB-KF, the measurement noise variance was adaptively updated using innovation statistics with a forgetting factor of ρ=0.98. A relatively large forgetting factor was adopted to ensure smooth noise variance adaptation and avoid excessive sensitivity to individual innovation samples. For the M-estimation-based robust KF, the Huber weighting function was used to reduce the influence of abnormal observations. The tuning constant was set to c=1.345, which is a commonly used setting in Huber-type robust estimation. The proposed method used the same parameter settings as those described in Section 3. No method-specific parameter tuning was performed to favor a particular algorithm; therefore, the comparison mainly reflects the difference in filtering mechanisms under the same state model, process noise covariance, initial conditions, and measured input sequence. The main parameter settings used in the comparison are summarized in Table 5.

The quantitative comparison results are shown in Table 6. Among the compared filtering methods, the standard KF gives a standard deviation of 24.059 ps. By adaptively updating the measurement noise variance, the VB-KF reduces the standard deviation to 22.667 ps. The M-estimation robust KF further reduces the standard deviation to 19.998 ps, indicating its ability to suppress abnormal observations and heavy-tailed disturbances. Compared with these representative methods, the proposed method achieves the lowest standard deviation of 17.681 ps.

**Figure 12:**
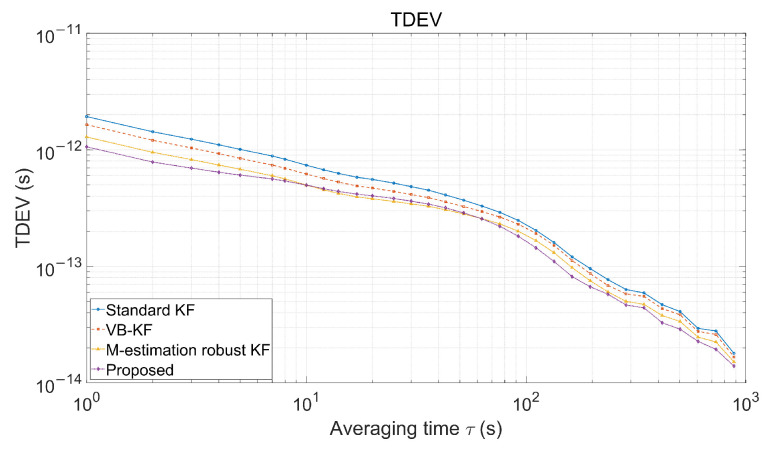
TDEV comparison of representative Kalman filtering methods using the same measured 1PPS time-offset sequence.

Figure 12 shows the TDEV comparison of the filtered 1PPS time-offset sequences obtained by the four Kalman filtering-based methods. To clearly distinguish the stability differences among the filtered results, the raw observation is not included in this figure. Under the same measured input sequence, the proposed method achieves the lowest TDEV at the selected averaging times of 1 s, 10 s, 100 s, and 800 s.

Overall, the proposed method shows better fluctuation suppression performance than the two representative adaptive and robust Kalman filtering methods. Compared with the VB-KF, the proposed method reduces the standard deviation from 22.667 ps to 17.681 ps; compared with the M-estimation robust KF, it further reduces the standard deviation from 19.998 ps to 17.681 ps. These results indicate that the combination of adaptive observation noise estimation and innovation consistency-based covariance regulation is more effective for the measured free-space laser time synchronization data than using adaptive noise estimation or robust innovation weighting alone. This combined mechanism enables the filter to adjust the measurement weight while maintaining sufficient tracking capability under time-varying link conditions. It should be emphasized that this offline comparison was used to evaluate the relative performance of different filtering algorithms under the same measured input sequence, and is not intended to replace the real-time experimental results reported in Section 4.3.

### 4.5. Ranging Results

**Figure 13:**
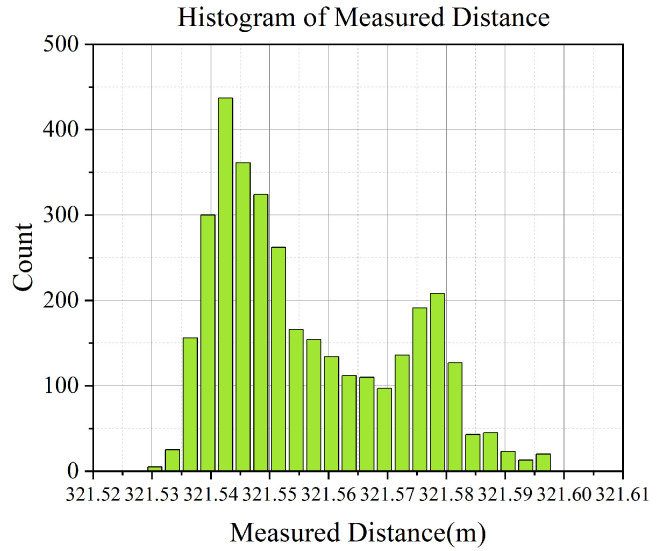
Statistical histogram of the ranging results obtained from the bidirectional free-space laser link.

In the established free-space laser experimental link, round-trip propagation delay measurements based on bidirectional timestamp exchange were further utilized to perform distance estimation. As described previously, the one-way free-space distance is approximately 160 m and the reflected signal forms a round-trip ranging path, corresponding to an equivalent ranging distance of about 321 m. Figure 13 presents the statistical histogram of the ranging results. The estimated distances are mainly distributed within the range of 321.53 m to 321.60 m, with a compact distribution and no evident outliers. Statistical analysis shows that the mean estimated distance is 321.556102 m and the ranging standard deviation is 0.015152 m (15.152 mm), demonstrating that the proposed system achieves centimeter-level ranging precision under free-space conditions.

**Figure 14:**
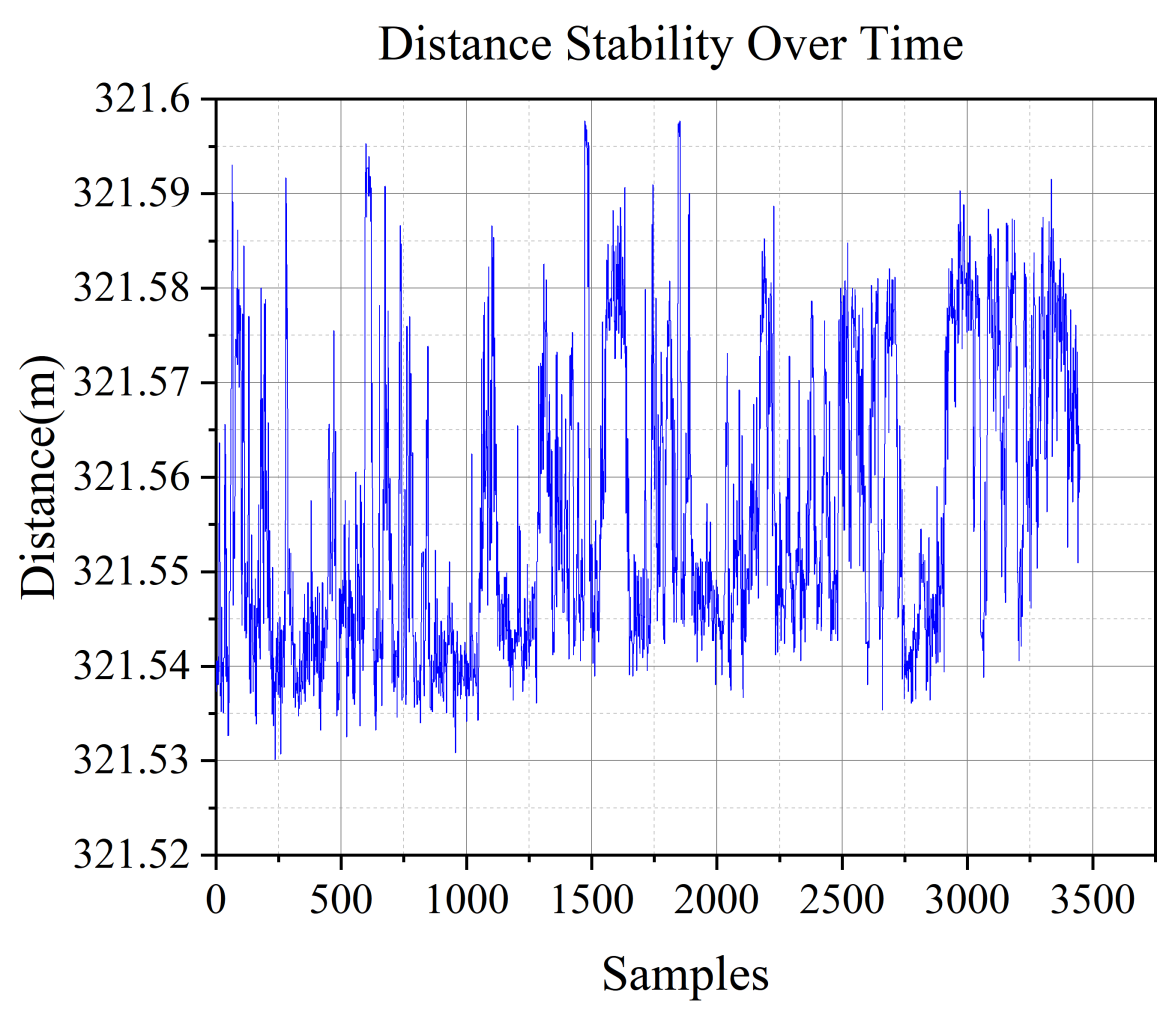
Time-domain evolution of the estimated ranging distance obtained from the bidirectional free-space laser link.

Figure 14 shows the time-domain evolution of the ranging results. Over the observation interval, the estimated distance varies continuously without abrupt jumps or discontinuities, while a slow drift trend can also be observed. The slow variation may be related to residual timing drift, environmental fluctuation, and the limited calibration accuracy of the practical free-space measurement link. Combined with the statistical distribution shown in Figure 13, it can be concluded that the proposed system achieves centimeter-level ranging precision over an equivalent free-space distance of approximately 321 m.

## 5. Conclusions

This paper has investigated high-precision time–frequency synchronization over free-space laser links, encompassing system implementation, algorithm design, and outdoor experimental validation. To address the non-Gaussian and highly fluctuating characteristics of time observation noise in free-space environments, we propose an improved Kalman filtering-based time synchronization method incorporating innovation consistency, then systematically evaluate the synchronization and ranging performance of the system using an equivalent free-space laser link with a total ranging distance of approximately 321 m.

Under the established experimental conditions, the system operates in a stable manner; the raw time offset measurements exhibit a time synchronization accuracy of 78.32 ps, accompanied by pronounced random excursions and a dispersed multimodal distribution. After applying the proposed improved Kalman filtering method based on innovation consistency, the time synchronization accuracy was significantly enhanced to 45.64 ps, corresponding to an improvement of approximately 41%. Meanwhile, the distribution of the time offset converged from a multimodal and dispersed state to an approximately unimodal and concentrated distribution, demonstrating that the proposed method effectively suppresses observation noise under free-space laser link conditions.

From the perspective of time stability, the time deviation (TDEV) of the time offset before filtering was approximately $7.14\times 10^{-11}$ s at an averaging time of τ=1 s, which was reduced to $4.33\times 10^{-11}$ s after filtering. At a longer averaging time of τ=800 s, the TDEV decreased from $4.20\times 10^{-12}$ s to $7.01\times 10^{-13}$ s. The overall TDEV curve after filtering remained consistently lower than that of the raw data, confirming the effectiveness of the proposed method in improving time synchronization stability over the tested averaging-time range.

In terms of ranging performance, free-space laser ranging experiments based on bidirectional timestamp exchange demonstrated that, under an equivalent ranging distance of approximately 321 m, the system achieved an average estimated distance of 321.556102 m with a ranging standard deviation of 15.152 mm. These results verify the feasibility of centimeter-level ranging under the present experimental conditions.

In summary, the experimental results demonstrate that, under the present ground-based free-space link configuration, filtering-based processing of time synchronization observations can improve the time synchronization accuracy by more than 40% without disturbing the internal clock operation of the system. The system also showed stable ranging performance in the integrated time–frequency synchronization and ranging experiment. The present work should be regarded as a ground-based verification of the proposed system and method. The experimental link was implemented over a free-space path of approximately 160 m with a reflector, corresponding to a round-trip equivalent distance of about 321 m. This configuration provides a controllable test environment for evaluating synchronization, filtering, and ranging performance. In future work, free-space links with longer distance and dynamic variation will be further investigated, with particular attention to the effects of Doppler shift, pointing fluctuation, platform vibration, thermal variation, received-power fluctuation, and other disturbances related to the space environment.

## Figures and Tables

**Table 1:** Main implementation parameters of the proposed filter.

Parameter	Value Used in Experiments
*p*	0.998
$Q_{\theta }$	$1.0\times 10^{-28}$
$Q_{\alpha }$	$5.0\times 10^{-27}$
β	0.3
γ	0.1
$\lambda_{\max}$	10
Confidence level	95%
$\chi_{0.95,2}^{2}$	5.991

**Table 2:** Pseudocode of the proposed innovation-adaptive Kalman filtering method.

Step	Operation
Input	Measured 1PPS time-offset sequence $\theta_{k}^{\left(m\right)}$, sampling interval *T*, parameters *p*, β, γ, $\lambda_{\max}$, initial state ${\hat{x}}_{0}$, initial covariance $P_{0}$, and process noise covariance *Q*.
Initialization	Set ${\hat{x}}_{0}={\left[\theta_{0},\alpha_{0}\right]}^{T}$, $P_{0}$, initial observation-noise variance ${\hat{\sigma }}_{\theta ,0}^{2}$, and recursive mean ${\bar{\theta }}_{0}$.
1	Perform state prediction according to the state-space model: ${\hat{x}}_{k}^{-}=F{\hat{x}}_{k-1}$ and $P_{k}^{-}=FP_{k-1}F^{T}+Q$.
2	Construct the observation vector from the measured time-offset data: $z_{k}={\left[\theta_{k}^{\left(m\right)},\alpha_{k}^{\left(m\right)}\right]}^{T}$, where $\alpha_{k}^{\left(m\right)}\approx \left(\theta_{k}^{\left(m\right)}-\theta_{k-1}^{\left(m\right)}\right)/T$.
3	Update the recursive mean ${\bar{\theta }}_{k}$ and the time-offset observation-noise variance ${\hat{\sigma }}_{\theta ,k}^{2}$ using the exponentially weighted recursive estimator.
4	Construct the correlated observation-noise covariance matrix $R_{k}$ according to the updated ${\hat{\sigma }}_{\theta ,k}^{2}$ and the correlation between $\theta_{k}^{\left(m\right)}$ and $\alpha_{k}^{\left(m\right)}$.
5	Compute the innovation vector and its covariance: $\nu_{k}=z_{k}-H{\hat{x}}_{k}^{-}$ and $S_{k}=HP_{k}^{-}H^{T}+R_{k}$. Then calculate the normalized innovation squared: ${\mathrm{NIS}}_{k}=\nu_{k}^{T}S_{k}^{-1}\nu_{k}$.
6	Perform innovation consistency checking. If ${\mathrm{NIS}}_{k}>\chi_{0.95,2}^{2}$, calculate $\lambda_{k}=1+\gamma \max\left(0,{\mathrm{NIS}}_{k}/\chi_{0.95,2}^{2}-1\right)$ and limit it by $\lambda_{\max}$; then inflate the predicted covariance as $P_{k}^{-}\leftarrow \lambda_{k}P_{k}^{-}$. Otherwise, keep $P_{k}^{-}$ unchanged.
7	Recalculate the innovation covariance using the adjusted predicted covariance and compute the Kalman gain: $S_{k}=HP_{k}^{-}H^{T}+R_{k}$ and $K_{k}=P_{k}^{-}H^{T}S_{k}^{-1}$.
8	Perform the primary measurement update using the 1PPS-based observation: ${\hat{x}}_{k}={\hat{x}}_{k}^{-}+K_{k}\nu_{k}$ and $P_{k}=\left(I-K_{k}H\right)P_{k}^{-}$.
9	Output the current estimates of the time offset ${\hat{\theta }}_{k}$ and frequency offset ${\hat{\alpha }}_{k}$, and use ${\hat{x}}_{k}$ and $P_{k}$ as the prior information for the next sampling instant.

**Table 3:** Approximate timing error budget related to the measured 1PPS offset.

Timing Term	Nature	Approximate Value/Bound	Relation to Filtering Result
Cable, connector, transceiver and PHY delay	Fixed calibrated delay	ps-level calibration uncertainty	Affects absolute timing bias; not directly reduced by filtering.
Optical terminal fixed delay	Fixed link delay	Included in system-level fixed-delay calibration	Affects absolute timing bias; not directly reduced by filtering.
Oscilloscope timing measurement uncertainty	External measurement noise	≈3 ps RMS	Minor random measurement contribution in the 1PPS offset sequence.
Residual clock-induced fluctuation	Slow time-varying fluctuation	Tens of ps RMS	Contributes to slow drift and residual time variation; can be suppressed in state estimation.
1PPS generation and output jitter	Random short-term jitter	≈20–50 ps RMS	Directly contributes to measured 1PPS offset fluctuation; can be suppressed in estimation.
Free-space optical-link fluctuation	Random/time-varying link disturbance	≈50–70 ps RMS	Dominant contributor to measured 1PPS offset fluctuation; can be suppressed in estimation.

**Table 4:** GMM fitting parameters of the detrended phase noise.

Component	Mean (°)	Std. Dev.	Weight
1	0.71582	0.84540	0.338
2	−0.78834	1.12572	0.186
3	1.71672	0.88621	0.083
4	−1.69702	0.85997	0.131
5	−0.06196	1.16629	0.263

**Table 5:** Main parameter settings used in the comparison with representative Kalman filtering-based methods.

Item	Parameter	Value
Common state model	Sampling interval *T*	1 s
Common state model	Decay factor *p*	0.998
Process noise	$Q_{\theta }$	$1.0\times 10^{-28}$
Process noise	$Q_{\alpha }$	$5.0\times 10^{-27}$
VB-KF	Forgetting factor ρ	0.98
M-est. robust KF	Huber constant *c*	1.345

**Table 6:** Offline comparison with representative Kalman filtering methods using the same measured 1PPS time-offset sequence.

Method	Std. Dev. (ps)	TDEV@1 s (s)	TDEV@10 s (s)	TDEV@100 s (s)	TDEV@800 s (s)
Standard KF	24.059	$1.9267\times 10^{-12}$	$7.3771\times 10^{-13}$	$2.2740\times 10^{-13}$	$2.2613\times 10^{-14}$
VB-KF	22.667	$1.6442\times 10^{-12}$	$6.1925\times 10^{-13}$	$2.1234\times 10^{-13}$	$2.1112\times 10^{-14}$
M-est. robust KF	19.998	$1.2884\times 10^{-12}$	$4.9893\times 10^{-13}$	$1.8456\times 10^{-13}$	$1.8636\times 10^{-14}$
Proposed	17.681	$1.0607\times 10^{-12}$	$4.9804\times 10^{-13}$	$1.6411\times 10^{-13}$	$1.6588\times 10^{-14}$

## Data Availability

All data can be provided upon reasonable request to the corresponding author.

## Abbreviations

The following abbreviations are used in this manuscript:

**Symbol**	**Definition**
$x_{k}$	State vector at the *k*-th sampling instant
$\theta_{k}$	Time-offset state between the master and slave nodes
$\alpha_{k}$	Frequency-offset state between the master and slave clocks
*T*	Sampling interval
*p*	Decay factor of the frequency-offset state
F	State transition matrix
$\omega_{k}$	Process noise vector at the *k*-th sampling instant
Q	Process noise covariance matrix
$z_{k}$	Observation vector at the *k*-th sampling instant
$\theta_{k}^{\left(m\right)}$	Measured time-offset observation
$\alpha_{k}^{\left(m\right)}$	Frequency-offset observation constructed from adjacent time-offset measurements
H	Observation matrix
$v_{k}$	Observation noise vector
R	Observation noise covariance structure for a given time-offset noise variance
$R_{k}$	Time-varying observation noise covariance matrix at the *k*-th sampling instant
$\nu_{k}$	Innovation vector
$S_{k}$	Innovation covariance matrix
${\mathrm{NIS}}_{k}$	Normalized innovation squared at the *k*-th sampling instant
$\lambda_{k}$	Covariance inflation factor
$\lambda_{\max}$	Upper bound of the covariance inflation factor
β	Smoothing factor of the recursive observation noise estimator
γ	Fading-strength parameter for covariance inflation
1PPS	One-pulse-per-second
CDR	Clock and data recovery
DDMTD	Digital dual-mixer time difference
EDFA	Erbium-doped fiber amplifier
GMM	Gaussian mixture model
KF	Kalman filter
NIS	Normalized innovation squared
PD	Photodetector
PTP	Precision time protocol
TDEV	Time deviation
